# Toxicity of Metals to a Freshwater Snail, *Melanoides tuberculata*

**DOI:** 10.1100/2012/125785

**Published:** 2012-04-24

**Authors:** M. Shuhaimi-Othman, R. Nur-Amalina, Y. Nadzifah

**Affiliations:** School of Environmental and Natural Resource Sciences, Faculty of Science and Technology, National University of Malaysia (UKM), Selangor, 43600 Bangi, Malaysia

## Abstract

Adult freshwater snails *Melanoides tuberculata* (Gastropod, Thiaridae) were exposed for a four-day period in laboratory conditions to a range of copper (Cu), cadmium (Cd), zinc (Zn), lead (Pb), nickel (Ni), iron (Fe), aluminium (Al), and manganese (Mn) concentrations. Mortality was assessed and median lethal times (LT_50_) and concentrations (LC_50_) were calculated. LT_50_ and LC_50_ increased with the decrease in mean exposure concentrations and times, respectively, for all metals. The LC_50_ values for the 96-hour exposures to Cu, Cd, Zn, Pb, Ni, Fe, Al, and Mn were 0.14, 1.49, 3.90, 6.82, 8.46, 8.49, 68.23, and 45.59 mg L^−1^, respectively. Cu was the most toxic metal to *M. tuberculata*, followed by Cd, Zn, Pb, Ni, Fe, Mn, and Al (Cu > Cd > Zn > Pb > Ni > Fe > Mn > Al). Metals bioconcentration in *M. tuberculata* increases with exposure to increasing concentrations and Cu has the highest accumulation (concentration factor) in the soft tissues. A comparison of LC_50_ values for metals for this species with those for other freshwater gastropods reveals that *M. tuberculata* is equally sensitive to metals.

## 1. Introduction

Metals are released from both natural sources and human activity. The impact of metals on the environment is an increasing problem worldwide. The impact of metals on aquatic ecosystems is still considered to be a major threat to organisms health due to their potential bioaccumulation and toxicity to many aquatic organisms. Although metals are usually considered as pollutants, it is important to recognize that they are natural substances. Zinc, for example, is an essential component of at least 150 enzymes; copper is essential for the normal function of cytochrome oxidase; iron is part of the haemoglobin in red blood cells; boron is required exclusively by plants [1]. Malaysia, as a developing country, is no exception and faces metals pollution caused especially by anthropogenic activities such as manufacturing, agriculture, sewage, and motor vehicle emissions [2–5]. Metals are nonbiodegradable. Unlike some organic pesticides, metals cannot be broken down into less harmful components. Managing metal contamination requires an understanding of the concentration dependence of toxicity. Dose-response relationships provide the basis for the assessment of hazards and risks presented by environmental chemicals. Toxicity testing is an essential tool for assessing the effect and fate of toxicants in aquatic ecosystems and has been widely used as a tool to identify suitable organisms as a bioindicator and to derive water quality standards for chemicals. There are many different ways in which toxicity can be measured, and most commonly the measure (end point) is death [1, 6, 7]. Metals research in Malaysia, especially using organisms as a bioindicator, is still scarce. Therefore, it is important to conduct studies with local organisms that can be used to gain data on metal toxicity, to determine the organism's sensitivity and to derive a permissible limit for Malaysian's water that can protect the local aquatic communities.

The freshwater molluscs of the Malaysian region are common, and most extant species are relatively easy to collect. The snails are rich fauna, while bivalve are the second. More than 150 aquatic nonmarine mollusc species have been recorded from the Malaysian region. *Melanoisdes tuberculata* (Müller 1774) is from class Gastropoda with shells higher than wide (elongate), conical, usually light brown in colour, and it is a cosmopolitan species [8]. *M. tuberculata* is a species of freshwater snail with an operculum, a parthenogenetic, aquatic gastropod mollusc in the family Thiaridae. The average shell length is about 20–27 mm and this species is native to subtropical and tropical northern Africa and southern Asia (Indo-Pacific region, Southern Asia, Arabia, and northern Australia), but they have established populations throughout the globe. The snail has an operculum that can protect it from desiccation and can remain viable for days on dry land [9]. It is a warm-climate species, prefers a temperature range of 18 to 32°C, and is primarily a burrowing species that tends to be most active at night. This snail feeds primarily on algae (microalgae) and acts as an intermediate host for many digenetic trematodes. *M. tuberculata* is a viviparous, gonochoric species with polyploid strains that reproduces by apomictic parthenogenesis. Because meiosis usually does not occur, offspring are identical to their mother. Females can be recognized by their greenish coloured gonads while males have reddish gonads. Under good conditions, females will produce fertilized eggs that are transferred to a brood pouch where they remain until they hatch. *M. tuberculata* will begin reproducing at a size as small as 5 to 10 mm in length and broods may contain over seventy offspring embryos which develop in the mother [10–12].

Molluscs have long been regarded as promising bioindicator and biomonitoring subjects. They are abundant in many terrestrial and aquatic ecosystems, being easily available for collection. They are highly tolerant to many pollutants and exhibit high accumulations of them, particularly heavy metals [13, 14]. Little information exists in the literatures concerning the toxic effects of metals for this snail. So far, only a few studies have been reported on metal toxicity to *M. tuberculata* [15, 16] and most of the studies were on the accumulation of metals [14, 17, 18]. Therefore, the purpose of this study was to determine the acute toxicity of eight metals (Cu, Cd, Zn, Pb, Ni, Fe, Al, and Mn) to the freshwater mollusc *M. tuberculata* and to examine the bioconcentration of these metals in the body after four days of exposure.

## 2. Materials and Methods

Snails *M. tuberculata* were collected from canals in the university in Bangi, Selangor, Malaysia. Identification of the species was based on Panha and Burch [8]. Prior to toxicity testing, the snails were acclimatized for one week under laboratory conditions (28–30°C with 12 h light :12 h darkness) in 50-L stocking tanks using dechlorinated tap water (filtered by several layers of sand and activated carbon; T.C. Sediment Filter (TK Multitrade, Seri Kembangan, Malaysia)) aerated through an air stone. During acclimation the snails were fed on lettuce. The standard stock solution (100 mg L^−1^) of Cu, Cd, Zn, Pb, Ni, Fe, Al, and Mn was prepared from analytical grade metallic salts of CuSO_4_·5H_2_O, CdCl_2_·2.5H_2_O, ZnSO_4_·7H_2_O, Pb(NO_3_)^2^, NiSO_4_·6H_2_0, FeCl_3_, Al_2_(SO_4_)_3_·18H_2_O, and MnSO_4_·H_2_O, respectively (Merck, Darmstadt, Germany). The stock solutions were prepared with deionized water in 1 L volumetric flasks. Acute Cu, Cd, Zn, Pb, Ni, Fe, Al, and Mn toxicity experiments were performed for a four-day period using adult snails (shell length approximately 1.5–2.0 cm, mean wet weight 22.5 ± 1.6 mg) obtained from stocking tanks. Following a range finding test, five Cu, Cd, Zn, Pb, Ni, Fe, Al, and Mn nominal concentrations were chosen (Table 1). Metal solutions were prepared by dilution of a stock solution with dechlorinated tap water. A control with dechlorinated tap water only was also used. The tests were carried out under static conditions with renewal of the solution every two days. Control and metal-treated groups each consisted of two replicates of five randomly allocated snails in a 500 mL glass beaker containing 400 mL of the appropriate solution. No stress was observed for the snails in the solution, indicated by 100% survival for the snails in the control water until the end of the study. A total of 10 animals per treatment/concentration were used in the experiment and a total of 410 animals were employed in the investigation [42, 43]. Samples of water for metal analysis taken before and immediately after each solution renewal were acidified to 1% with ARISTAR nitric acid (65%) (BDH Inc, VWR International Ltd., England) before metal analysis by flame or furnace Atomic Absorption Spectrophotometer (AAS-Perkin Elmer model AAnalyst800, Massachusetts, USA) depending on the concentrations.

During the toxicity test, the snails were not fed. The experiments were performed at room temperature of 28–30°C with photoperiod 12 h light : 12 h darkness, using fluorescent lights (334–376 lux). Water quality parameters (pH, conductivity, and dissolved oxygen) were measured every two days using portable meters (model Hydrolab Quanta, Hach, Loveland, USA) and water hardness samples were fixed with ARISTAR nitric acid and measured by flame atomic absorption spectrophotometer (AAS—Perkin Elmer model AAnalyst 800). Mortality was recorded every 3 to 4 hours for the first two days and then at 12 to 24 hour intervals throughout the rest of the test period. The criterion used to determine mortality were failure to respond to gentle physical stimulation. The death was further confirmed by putting the snail on the glass petri dish for few minutes and if it did not show any movement, it was considered dead. Any dead animals were removed immediately.

At the end of day four, the live snails were used to determine bioconcentration of the metals in the whole body (soft tissues) according to the concentrations used. The snails were cleaned with dechlorinated tap water, and soaked in boiling water for approximately 3 min. Tissues of the molluscs were removed from the shell, rinsed with deionized water, and each sample contained three replicates of three to five animals in a glass test tube (depending on how many live animals were left) and was oven-dried (80°C) for at least 48 hours before being weighed [14]. Each replicate was digested (whole organism) in 1.0 mL ARISTAR nitric acid (65%) in a block thermostat (80°C) for 2 hours. Upon cooling, 0.8 mL of hydrogen peroxide (30%) was added to the solutions. The test tubes were put back on the block thermostat for another 1 hour until the solutions became clear. The solutions were then made up to 25 mL with the addition of deionized water in 25 mL volumetric flasks. Efficiency of the digestion method was evaluated using mussel and lobster tissue reference material (SRM 2976 and TORT-2, National Institute of Standard and Technology, Gaithersburg, USA and National Research Council Canada, Ottawa, Ontario, Canada, resp.). Efficiencies obtained were within 10% of the reference values. To avoid possible contamination, all glassware and equipment used were acid-washed (20% HNO_3_) (Dongbu Hitek Co. Ltd., Seoul, Korea, 68%), and the accuracy of the analysis was checked against blanks. Procedural blanks and quality control samples made from standard solutions for Cu, Cd, Zn, Pb, Ni, Fe, Al, and Mn (Spectrosol, BDH, England) were analyzed in every ten samples in order to check for sample accuracy. Percentage recoveries for metals analyses were between 85–105%.

Median lethal times (LT_50_) and concentrations (LC_50_) for the snails exposed to metals were calculated using measured metal concentrations. FORTRAN programs based on the methods of Litchfield [44] and Litchfield and Wilcoxon [45] were used to compute the LT_50_ and LC_50_. Data were analyzed using time/response (TR) and concentration/response (CR) methods by plotting cumulative percentage mortality against concentration and time, respectively, on logarithmic-probit paper. Concentration factors (CFs) were calculated for whole animals as the ratio of the metals concentrations in the tissues to the metals concentration measured in the water.

## 3. Results and Discussion

In all data analyses, the actual (measured concentration) rather than nominal Cu, Cd, Zn, Pb, Ni, Fe, Al, and Mn concentrations were used (Table 1). The mean water quality parameters measured during the test were pH 6.68 ± 0.22, conductivity 180.0 ± 46.0 *μ*S cm^−1^, dissolved oxygen 6.1 ± 0.27 mg L^−1^, and total hardness (Mg^2+^and Ca^2+^) 18.72 ± 1.72 mg L^−1^ as CaCO_3_.

**Figure 1:**
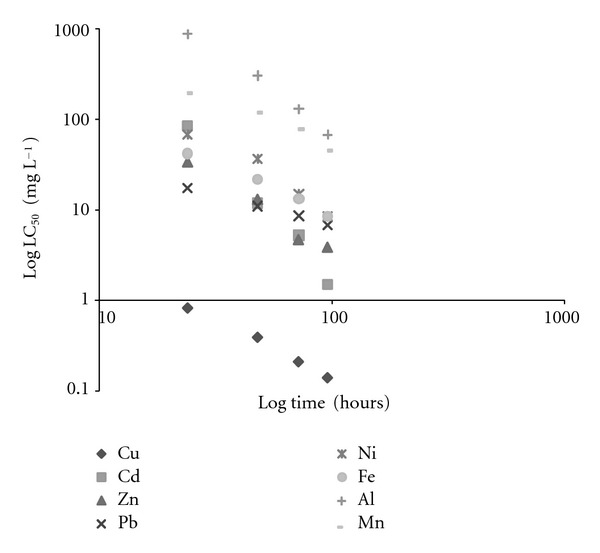
The relationship between median lethal concentration (LC_50_) and exposure times for *M. tuberculata*.

**Figure 2:**
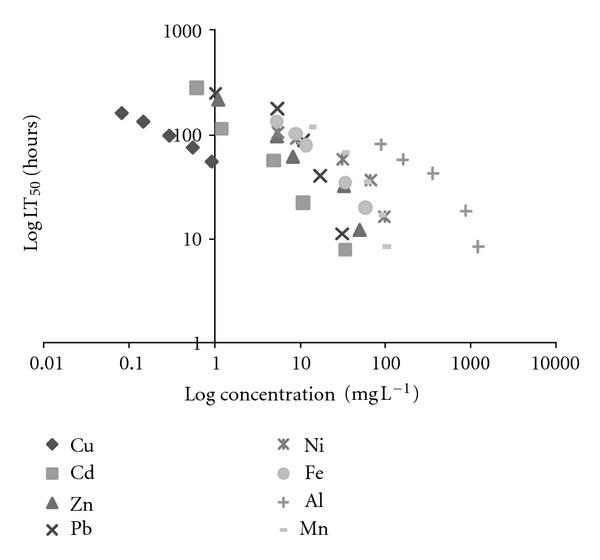
The relationship between median lethal time (LT_50_) and exposure concentrations for *M. tuberculata*.

One hundred percent of control animals maintained in dechlorinated tap water survived throughout the experiment. The median lethal times (LT_50_) and concentrations (LC_50_) increased with a decrease in mean exposure concentrations and times, respectively, for all metals (Tables 1 and 2). However, the lethal threshold concentration could not be determined since the toxicity curves (Figures 1 and 2) did not become asymptotic to the time axis within the test period. Figures 1 and 2 show that Cu was the most toxic metal to *M. tuberculata*, followed by Cd, Zn, Pb, Ni, Fe, Mn, and Al. Other studies show different trends of toxicity with different snails. According to Luoma and Rainbow [7] the rank order of toxicity of metals will vary between organisms. With *Lymnaea luteola*, Khangarot and Ray [28, 30] showed that the order of toxicity was Cd > Ni > Zn; with *Viviparus bengalensis*, Gupta et al. [27] and Gadkari and Marathe [34] found that the order of toxicity was Zn > Cd > Pb > Ni; and with *Juga plicifera*, Nebeker et al. [20] found that Cu was more toxic than Ni.

The present study showed that LC_50_s for 48 and 96 hours of Cu, Cd, Zn, Pb, Ni, Fe, Al, and Mn were 0.39, 11.85, 13.15, 10.99, 36.46, 21.78, 306.89, and 120.43 mg L^−1^, and 0.14, 1.49, 3.90, 6.82, 8.46, 8.49, 68.23 and 45.59 mg L^−1^, respectively (Table 1). A few studies had reported on the acute toxicity of metals to *M. tuberculata*. Bali et al. [15] and Mostafa et al. [16] showed that 96 h-LC_50_ of Cu to *M. tuberculata* were 0.2 and 3.6 mg L^−1^, respectively, which were higher than the present study. In comparison with other freshwater gastropods (Table 3), this study showed that in general LC_50_s for *M. tuberculata* were lower or similar compared to other freshwater snails. Direct comparisons of toxicity values obtained in this study with those in the literature were difficult because of differences in the characteristics (primarily water hardness, pH, and temperature) of the test waters. With similar water hardness (soft water) and using adult snails, Nebeker et al. [20] reported that 96 h-LC_50_ of Cu for *Fluminicola virens* was 0.08 mg L^−1^, and of Zn for *Physa Gyrina* was 1.27 mg L^−1^, which was lower than the present study. The toxicity reported by other studies (Table 3) differs from that reported in this study owing to the different species, ages, and sizes of the organisms as well as varied test methods (water quality and water hardness) as this can affect toxicity [46–49]. In the present study, the water hardness used was considered low (18.7 mg L^−1^ CaCO_3_), and the water was categorized as soft water (<75 mg L^−1^ as CaCO_3_).

In comparison with other taxa, *M. tuberculata* shows less sensitivity to metals. LC_50_s reported for other taxa from this laboratory such as Crustacea (prawn *Macrobrachium lanchesteri* [50] and ostracod *Stenocypris major* [51]), fish (*Rasbora sumatrana* and *Poecilia reticulata* [52]), and Annelida (*Nais elinguis* [53]) were lower than the LC_50_ values of *M. tuberculata* in the present study. Von Der Ohe and Liess [54] showed that 13 taxa belonging to Crustacea were among the most sensitive to metal compounds and concluded that taxa belonging to Crustacea are similar to one another and to *Daphnia magna* in terms of sensitivity to organics and metals and that Molluscs have an average sensitivity to metals. Mitchell et al. [9] reported that the snail has a tightly sealing operculum that allows it to withstand desiccation and apparently also increases its tolerance to chemicals.

**Figure 3:**
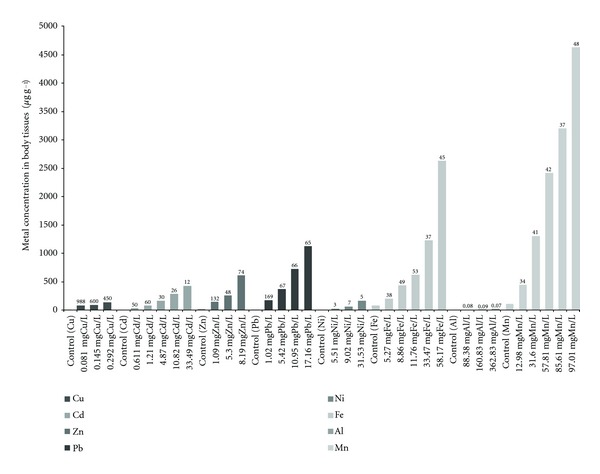
Bioconcentration of Cu, Cd, Zn, Pb, Ni, Fe, Al, and Mn (mean) in *M. tuberculata* soft tissues (*μ*g g^−1^ dry weight) after a four-day exposure to different concentrations of Cu, Cd, Zn, Pb, Ni, Fe, Al, and Mn. Concentration factor (CF) is indicated at the top of each bar.

Bioconcentration of Cu, Cd, Zn, Pb, Ni, Fe, Al, and Mn in surviving *M. tuberculata* is as shown in Figure 3. Bioconcentration data for live snails were obtained from five Cd (0.61, 1.21, 4.87, 10.82 and 33.49 mg L^−1^), Fe (5.27, 8.86, 11.76, 33.47, and 58.17 mg L^−1^), and Mn (12.98, 31.60, 57.81, 85.61 and 97.01 mg L^−1^) concentration exposures; four Pb (1.02, 5.42, 10.95 and 17.16 mg L^−1^) concentration exposures; three Cu (0.081,0145 and 0.292 mg L^−1^), Zn (1.09, 5.30 and 8.19 mg L^−1^), Ni (5.51, 9.02 and 31.53 mg L^−1^), and Al (88.38, 160.83 and 362.83 mg L^−1^) concentration exposures. In general, the Cu, Cd, Pb, Zn, Ni, Fe, Al, and Mn bioconcentration in *M. tuberculata* increases with increasing concentration exposure. Similar results were reported by Moolman et al. [18] on Cd and Zn accumulation by two freshwater gastropods (*M. tuberculata* and *Helisoma duryi*). Hoang and Rand [55] showed that whole body Cu concentration of juvenile apple snails (*Pomacea paludosa*) was significantly correlated with soil and water Cu concentrations. In other experiments, Hoang et al. [56] showed that whole body Cu concentrations of juvenile snails (*P. paludosa*) increased with exposure time and concentration and reached a plateau (saturation) after 14 days of exposure. These results are in agreement with the statement of Luoma and Rainbow [7] who state that the uptake of trace metals from solution by an aquatic organism is primarily concentration dependent. The higher the dissolved concentration of the trace metal, the higher the uptake of the metal from solution into the organism will be, until the uptake mechanism becomes saturated. 

The present study shows that in general the highest concentration factor (CF) was noted for Cu (988), Pb (169), and Zn (132), and the lowest CF was for Al (0.07) (Figure 3). Similar results were reported by Lau et al. [14] who reported that *M. tuberculata* collected from the wild (Sarawak River) accumulated higher amounts of Cu, Zn, and As in the soft tissues compared to other metals. Adewunmi et al. [17] showed that Cu, Pb, and Cd were the highest metal accumulated in tissues of freshwater snails in dams and rivers in southwest Nigeria, and metal concentrations in the snails were varied with the seasons, especially for Cu which was higher in the dry season compared to the rainy season. According to Luoma and Rainbow [7] the factors that affect the rate of uptake of metals affect the toxicity of metal. This is in agreement with the results from the present study which shows that Cu, which was the most toxic to the snail, also has the highest CF in the soft tissues of *M. tuberculata*. In explaining the toxicity of Cu, Hoang and Rand [55] demonstrate that the potential toxicity of Cu carbonate to snails may be explained by the carbonate content in the snails. The carbonate requirement for snails is more than for fish because snails require it for shell development. Copper may enter snails as Cu carbonate. After entering snails, Cu carbonate may be disassociated through biological and chemical reactions. Carbonate would be available for shell development and Cu would be accumulated in soft tissue. Hoang et al. [56] also reported that with the juvenile apple snail (*Pomacea paludosa*), most of the accumulated Cu was located in soft tissue (about 60% in the viscera and 40% in the foot) and the shell contained <4% of the total accumulated copper. However, a comparison of the uptake rate in aquatic organisms showed that in general the order of the uptake rate constant is Ag > Zn > Cd > Cu > Co > Cr > Se [7]. This discrepancy is probably due to short time of exposure (four days) to metals in this study. Other factors which may influence the bioaccumulation of heavy metals in aquatic organisms has been suggested, such as their feeding habit [57], growth rate and age of the organism [14, 58], and the bioavailability of the metals, which greatly depends on hardness of water, pH, and the acid-volatile sulphide of the water [59]. Hoang and Rand [55] showed that the apple snails (*Pomacea paludosa*) accumulated more Cu from soil-water than from water-only treatments and this suggests that apple snails accumulate Cu from soil (-sediment)/water systems. Organisms with higher growth rates also usually have lower metal concentrations in their bodies as the rate of increase in the weight of its tissue and shell will be higher than the accumulated metals [14]. According to Lau et al. [14], the shell of *M. tuberculata* would be most suitable for monitoring Cu in the aquatic environment, which has an approximately thirtyfold magnification capability and with standard errors of less than 10%. Zn would be best monitored by using the shell of *M. tuberculata,* whose magnification capability was approximately 35 times and its error was at approximately 15%. Both tissue and shell of *M. tuberculata* could also be used for monitoring arsenic as it has good magnification capabilities with moderate irregularity approximately 23%. However, it is important to note that the Lau et al. [14] study was conducted in the field (long-term exposure), while the present study was conducted in the laboratory with short-term exposure, and differences in accumulation trend and strategies (higher accumulation in soft tissues or shell) may exist.

Aquatic molluscs possess very diverse strategies in the handling and storage of accumulated metals, which include being in the forms of metal-rich granules metallothioneins (MT) or metallothionein-like proteins [60–62]. Accumulation strategies of invertebrates vary intraspecifically between metals and interspecifically for the same metal in closely related organisms [62, 63]. Moolman et al. [18] showed that *M. tuberculata* had a much higher uptake of Zn in the Zn and in the mixed Cd/Zn exposures compared to *Helisoma duryi*, and Zn was readily accumulated with increasing metal concentrations. Lau et al. [14] also demonstrated that Zn concentrations in *M. tuberculata* were significantly higher than those in the molluscs *Brotia costula* and *Clithon* sp. The present study shows that the CF of Zn was higher than the Cd in the soft tissues of *M. tuberculata*. With the juvenile apple snail, Hoang et al. [56] showed that the snails accumulated Cu during the exposure phase and eliminated Cu during the depuration phase. Metals accumulated in animals can be stored without excretion leading to high body concentrations (accumulators), or the metal levels in the body can be maintained at a low constant body concentration (regulators) by balancing the uptake with controlled rates of excretion [64].

## 4. Conclusions

This study showed that *M. tuberculata *was equally sensitive to metals compared to other freshwater gastropods. Cu was the most toxic metal to *M. tuberculata* followed by Cd, Zn, Pb, Ni, Fe, Mn, and Al. A comparison of the bioconcentration of metals in soft tissues of *M. tuberculata* showed that among the eight metals studied; Cu, Pb, and Zn were the most accumulated and Al was least accumulated. *M. tuberculata* is widely distributed in urban and suburban areas which makes it easy to sample and very useful in ecotoxicology studies. This study indicates that *M. tuberculata* could be a potential bioindicator organism of metals pollution and in toxicity testing.

## Figures and Tables

**Table 1:** Median lethal times (LT_50_) for *M. tuberculata* exposed to different concentrations for Cu, Cd, Zn, Pb, Ni, Fe, Al, and Mn.

Nominal (and measured) concentration (mg L^−1^)	LT_50_ (h)	95% Confidence limits
Cu		
0.075 (0.081) 0.1 (0.145) 0.32 (0.292) 0.56 (0.549) 0.87 (0.915)	163.42 134.97 98.89 75.87 55.42	63.60–419.91 53.22–342.28 44.35–220.50 26.88–214.15 25.36–121.11

Cd		
0.56 (0.611) 1.0 (1.21) 5.6 (4.87) 10 (10.82) 32 (33.49)	283.44 114.89 57.21 22.34 7.82	85.46–940.12 52.78–250.09 30.29–108.05 11.03–45.27 4.63–13.23

Zn		
1.0 (1.09) 5.6 (5.30) 10 (8.19) 32 (32.45) 56 (49.60)	216.96 96.71 61.83 32.44 12.34	630.53–1541.63 52.82–177.09 39.85–95.94 22.13–47.55 8.25–18.45

Pb		
1.0 (1.02) 5.6 (5.42) 10 (10.95) 18 (17.16) 32 (31.18)	250.72 179.32 88.25 40.36 11.17	430.55–2057.69 38.42–837.02 30.24–257.71 13.91–117.15 6.71–18.57

Ni		
5.6 (5.51) 10 (9.02) 32 (31.53) 75 (67.11) 100 (97.84)	105.96 92.38 58.11 36.84 16.26	59.28–189.40 48.25–176.87 39.82–84.80 21.66–62.67 12.50–21.17

Fe		
5.6 (5.27) 8.7 (8.86) 10 (11.76) 32 (33.47) 56 (58.17)	134.97 102.06 79.72 34.71 20.04	53.22–342.28 40.17–259.29 28.18–225.53 13.36–90.15 7.5–53.51

Al		
56 (88.38) 100 (160.83) 320 (362.83) 560 (884.34) 1000 (1229.91)	80.87 57.91 42.75 18.57 8.40	111.48–58.66 87.09–38.50 65.99–27.70 40.94–8.42 18.52–3.81

Mn		
10 (12.98) 32 (31.60) 56 (57.81) 87 (85.61) 100 (97.01)	119.53 67.81 35.07 16.97 8.35	62.44–228.82 35.06–131.15 19.04–64.60 9.00–32.00 5.05–13.79

**Table 2:** Median lethal concentrations (LC_50_) for *M. tuberculata* at different exposure times for Cu, Cd, Zn, Pb, Ni, Fe, Al, and Mn.

Time (hour)	LC_50 _(mg L^−1^)	95% Confidence limits
Cu		
24 48 72 96	0.82 0.39 0.21 0.14	0.49–4.21 0.23–0.88 0.12–0.33 0.09–0.20

Cd		
24 48 72 96	85.03 11.85 5.24 1.49	13.94–518.57 2.70–51.99 0.96–28.43 0.34–6.53

Zn		
24 48 72 96	33.97 13.15 4.73 3.90	21.59–65.31 6.93–26.06 2.28–8.10 1.81–6.67

Pb		
24 48 72 96	17.39 10.99 8.57 6.82	12.06–29.47 6.04–19.68 4.37–14.79 2.89–12.67

Ni		
24 48 72 96	68.35 36.46 15.04 8.46	48.18–102.23 20.76–70.91 5.23–28.97 3.53–14.02

Fe		
24 48 72 96	42.12 21.78 13.29 8.49	25.74–133.99 10.52–88.85 4.09–29.47 1.58–15.25

Al		
24 48 72 96	880.78 306.89 130.22 68.23	553.91–2147.55 184.29–487.20 35.51–226.38 2.24–123.87

Mn		
24 48 72 96	194.52 120.43 78.35 45.59	112.85–335.27 58.08–249.72 36.20–169.56 20.17–103.04

**Table 3:** Comparison of LC_50_ values of freshwater gastropod *M. tuberculata* with other freshwater mollusc.

Metal	Species	Water hardness (mg L^−1^)	Live stage	Test duration	LC_50_ (mg L^−1^)	Reference
Copper	*M. tuberculata*	18.7	Adult	96 h	0.14	This study
	*M. tuberculata*			48 h	3.6	[16]
	*M. tuberculata*		Juvenile	24 h	0.2	[15]
	*B. glabrata*	44	Adult	48 h	0.18	[19]
	*F. virens*	21	Adult	96 h	0.08	[20]
	*J. plicifera*	21	Adult	96 h	0.015	[20]
	*B. glabrata*	100	—	96 h	0.04	[21]
	*P. paludosa*	68	60 d	96 h	0.14	[22]
	*P. jenkinsi*	—	Adult	96 h	0.08	[23]

Cadmium	*M. tuberculata*	18.7	Adult	96 h	1.49	This study
	*Amnicola *sp.	50	Adult	96 h	8.4	[24]
	*P. fontinalis *	—	—	96 h	0.08	[25]
	*A. hypnorum*	45	Adult	96 h	0.09	[26]
	*B. glabrata*	100	—	96 h	0.3	[21]
	*V. bengalensis*	180	—	96 h	1.2	[27]
	*L. luteola*	195	Adult	96 h	1.5	[28]

Zinc	*M. tuberculata *	18.7	Adult	96 h	3.90	This study
	*P. gyrina*	36	Adult	96 h	1.27	[20]
	*L. acuminata*	375	—	96 h	10.49	[29]
	*L. luteola*	195	Adult	96 h	11.0	[30]
	*V. bengalensis*	180	—	96 h	0.64	[27]
	*P. heterostropha*	20	Adult	96 h	1.11	[31]
	*P. heterostropha*	100	Adult	96 h	3.16	[31]

Lead	*M. tuberculata *	18.7	Adult	96 h	6.82	This study
	*L. emarginata*	150	—	48 h	14.0	[32]
	*E. livescens*	150	—	48 h	71.0	[32]
	*Filopaludina sp.*	—	Adult	96 h	190	[33]
	*V. bengalensis*	165	—	96 h	2.54	[34]
	*A. hypnorum*	60.9	—	96 h	1.34	[35]

Nickel	*M. tuberculata *	18.7	Adult	96 h	8.46	This study
	*Amnicola *sp.	50	Adult	96 h	14.3	[24]
	*J. plicifera *	59	Adult	96 h	0.24	[20]
	*L. luteola*	195	Adult	96 h	1.43	[28]
	*V. bengalensis*	180	—	96 h	9.92	[27]
	*L. acuminata*	375	—	96 h	2.78	[29]

Iron	*M. tuberculata*	18.7	Adult	96 h	8.49	This study
	*P. gyrina*	109	—	96 h	12.09	[36]
	*Planorbarius sp.*	—	—	48 h	7.32	[37]
	*S. libertina*	—	—	48 h	76.0	[38]

Aluminium	*M. tuberculata*	18.7	Adult	96 h	68.23	This study
	*Physa sp.*	47	—	96 h	55.5	[39]
	*A. limosa*	PH 3.5	—	96 h	1.0	[40]
	*A. limosa*	PH 4.5	—	96 h	0.40	[40]

Manganese	*M. tuberculata*	18.7	Adult	96 h	45.59	This study
	*B. globosus*	53	—	96 h	100.0	[41]
